# Consensus Analysis of Whole Transcriptome Profiles from Two Breast Cancer Patient Cohorts Reveals Long Non-Coding RNAs Associated with Intrinsic Subtype and the Tumour Microenvironment

**DOI:** 10.1371/journal.pone.0163238

**Published:** 2016-09-29

**Authors:** James R. Bradford, Angela Cox, Philip Bernard, Nicola J. Camp

**Affiliations:** 1 Sheffield Institute for Nucleic Acids (SInFoNiA), Department of Oncology and Metabolism, University of Sheffield, Sheffield, South Yorkshire, United Kingdom; 2 Department of Pathology, Huntsman Cancer Institute, University of Utah, Salt Lake City, Utah, United States; 3 Department of Internal Medicine, Huntsman Cancer Institute, University of Utah, Salt Lake City, Utah, United States; Washington State University, UNITED STATES

## Abstract

Long non-coding RNAs (lncRNAs) are emerging as crucial regulators of cellular processes and diseases such as cancer; however, their functions remain poorly characterised. Several studies have demonstrated that lncRNAs are typically disease and tumour subtype specific, particularly in breast cancer where lncRNA expression alone is sufficient to discriminate samples based on hormone status and molecular intrinsic subtype. However, little attempt has been made to assess the reproducibility of lncRNA signatures across more than one dataset. In this work, we derive consensus lncRNA signatures indicative of breast cancer subtype based on two clinical RNA-Seq datasets: the Utah Breast Cancer Study and The Cancer Genome Atlas, through integration of differential expression and hypothesis-free clustering analyses. The most consistent signature is associated with breast cancers of the basal-like subtype, leading us to generate a putative set of six lncRNA basal-like breast cancer markers, at least two of which may have a role in *cis*-regulation of known poor prognosis markers. Through *in silico* functional characterization of individual signatures and integration of expression data from pre-clinical cancer models, we discover that discordance between signatures derived from different clinical cohorts can arise from the strong influence of non-cancerous cells in tumour samples. As a consequence, we identify nine lncRNAs putatively associated with breast cancer associated fibroblasts, or the immune response. Overall, our study establishes the confounding effects of tumour purity on lncRNA signature derivation, and generates several novel hypotheses on the role of lncRNAs in basal-like breast cancers and the tumour microenvironment.

## Background

Remarkable progress over the last decade has challenged the idea that the human transcriptome is derived exclusively from protein-coding (PC) genes and a few specific non-coding RNAs. This so-called pervasive transcription is widespread, with some studies estimating that up to 90% of the genome is transcribed despite PC genes representing <2% of the total genomic sequence [1]. A major component of non-coding species consists of long non-coding RNAs (lncRNAs) defined as RNA of >200nt in length with no apparent coding capacity. LncRNAs function through a variety of mechanisms including remodelling of chromatin, transcriptional co-activation or -repression, protein inhibition, post-transcriptional modification, or decoy. They can regulate gene expression either transcriptionally or post-transcriptionally, acting either on the same locus (*cis*) or more distal sites (*trans*). For some lncRNAs, the act of transcription alone is sufficient to regulate their neighbouring genes by altering local chromatin state [2]. For others, subcellular specificity and splicing suggest a mature RNA molecule is required for function [3].

LncRNAs are now emerging as crucial regulators of cellular processes and diseases, and their aberrant transcription can lead to altered expression of target genes involved in cancer pathways and functions [4]. For example, over-expression of several prominent lncRNAs such as *HOTAIR* in colorectal and metastatic breast cancers [5][6][7], *PCAT1* in prostate cancer [8], and *MALAT1* in early-stage non small-cell lung cancer [9], has been linked to poor prognosis and tumour progression. Despite these advances, the vast majority of lncRNAs identified through large-scale efforts such as GENCODE [10] and MiTranscriptome [11] remain poorly understood. To address this gap, two recent genome-wide pan-cancer studies used integrative genomic approaches to assign putative function to several thousand lncRNAs [12][13]. Both studies incorporated the “guilt-by-association” strategy for *in silico* lncRNA function characterization, deriving a prediction based on a common expression pattern between the lncRNA and a biological process or pathway [14]. These and other studies also demonstrated the disease and tumour subtype specificity of lncRNAs [12][13][15][16]. Notably, lncRNA expression alone is sufficient to discriminate breast cancer samples based on hormone status and molecular intrinsic subtype [12][17][18], achieving greater specificity than PC genes [12].

In this work, we build on these subtype association studies by deriving breast cancer subtype-specific lncRNA signatures from two patient cohorts: The Cancer Genome Atlas (TCGA), and the Utah Breast Cancer Study (UBCS). First we evaluate signature consistency between the two datasets, and then determine the underlying cause of any disparity through “guilt-by-association” with PC genes, and integration of pre-clinical expression data. By doing so, our study reveals the influence of tumour purity on lncRNA signature derivation from patient samples, and proposes several lncRNAs whose expression is specific to cells in the breast tumour microenvironment.

## Results

The UBCS RNA-Seq dataset was derived from fresh frozen breast tissue samples obtained from 88 women who had surgery at the Huntsman Cancer Hospital from 2009–2012. These included tumour tissues from 69 breast cancer patients, of which 51 ER+ and 12 triple-negative breast cancer (TNBC) tumours were selected for this study. A mean of 18,704,489 reads were uniquely mapped to the human genome corresponding to a mapping success rate of 87% (S1 Table). To achieve consistency with UBCS, TCGA RNA-Seq reads across 271 ER+ and 68 TNBC patients were re-mapped using a similar protocol (see Methods), resulting in a mean of 67,741,640 reads uniquely mapped to the human genome and a mapping success rate of 86% (S2 Table).

### LncRNA expression generates clusters that correspond to hormone status and intrinsic subtype

**Fig 1:**
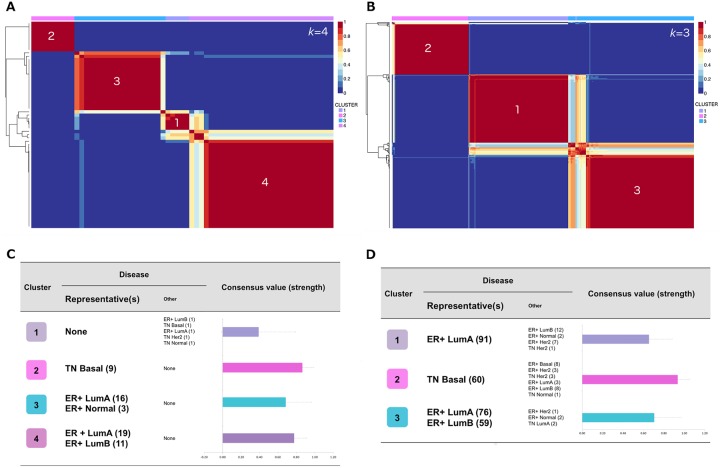
Application of non-negative matrix factorization (NMF) to optimal clustering of UBCS and TCGA lncRNA expression. A, consensus matrix at *k* = 4 for lncRNA expression across 63 UBCS samples. B, consensus matrix at *k* = 3 for lncRNA expression across 339 TCGA samples. C, contributing cancer types and mean consensus value of each UBCS cluster. D, contributing cancer types and mean consensus value of each TCGA cluster. “Representative” disease indicates the majority breast cancer subtype in the cluster, and numbers of models are given in brackets. Mean consensus value was computed from 200 runs of NMF.

We applied non-negative matrix factorization (NMF) [19][20] to cluster 932 and 588 of the most highly expressed ((mean FPKM + standard deviation (SD))>1.00) and variable (coefficient of variation (CV)>0.10) GENCODE [10] annotated lncRNAs across UBCS and TCGA cohorts respectively, and tested whether lncRNA expression signatures allow separation of breast cancer into distinct subtypes. Stable clusters were achieved at *k* = 4 (UBCS; Fig 1A) and *k* = 3 (TCGA; Fig 1B) where *k* denotes the number of clusters selected according to the procedure outlined in *Methods*. Model-to-cluster mappings for both UBCS and TCGA are given in S1 and S2 Tables respectively, and genes deemed as key drivers of the clustering (meta-genes) are listed in S3a (UBSC) and S3b (TCGA) Table.

Across both datasets, clusters broadly corresponded to breast cancer hormone status. Of the four UBCS clusters, clusters three and four consisted exclusively of ER+ tumours, and cluster two of TNBC tumours, with cluster one comprising a “mixed” theme of two ER+ and three TNBC tumours (Fig 1C; S4a Table). The two ER+ clusters were differentiated according to PAM50 intrinsic subtype [21] with luminal A tumours comprising the majority (16/19) of cluster three, and the remainder of the cluster including three normal-like tumours. Cluster four encompassed both ER+ luminal A (19/30) and luminal B (11/30) tumours. All TNBC tumours of cluster two corresponded to the basal-like subtype, whereas the three TNBC tumours in cluster one were classified as basal, HER2- and normal-like subtypes.

Clusters derived from TCGA followed a similar pattern (Fig 1D; S4b Table), with themes relating to ER+ luminal A (cluster one; 91/113 tumours), basal-like (cluster two; 60/86) and ER+ luminal A/B (cluster three; luminal A: 76/140, luminal B: 59/140). Whilst the majority of cluster two consisted of TNBC, all eight ER+ basal-like tumours were members of the cluster two, further suggesting separation was being driven by intrinsic subtype rather than hormone status. Note that no ER+ basal-like tumours were present in the UBCS dataset.

### A consensus set of basal breast cancer lncRNA markers

By comparing the cluster meta-genes of UBCS and TCGA (Table 1), we found significant overlap (*p*<0.001 by hyper-geometric test) between the TNBC basal clusters, and between the ER+ luminal A/B clusters (*p*<0.001). By contrast, very little overlap was observed between the ER+ luminal A cluster, and none of the 14 genes driving the UBCS mixed subtype cluster achieved the expression and variance criteria in the TCGA sample set.

**Table 1:** Comparison between meta-genes driving clustering of UBSC and TCGA tumour samples.

	Cluster (number of meta-genes)		UBCS (932 genes)			
			1 (14)	2 (17)	3 (12)	4 (28)
		Representative subtype	Mixed	TNBC basal-like	ER+ luminal A/Normal	ER+ luminal A/B
TCGA (588 genes)	1 (20)	ER+ luminal A	0	0	0	3
	2 (18)	Basal-like	0	9*	0	0
	3 (33)	ER+ luminal A/B	0	0	1	14*

*p<0.001

**Fig 2:**
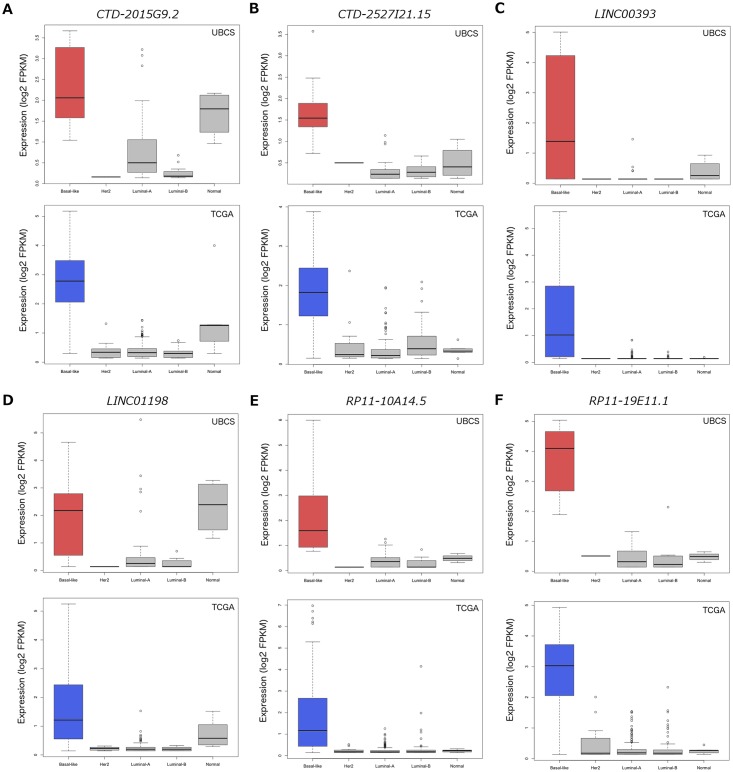
Comparison of lncRNA basal-like marker expression between breast cancer intrinsic subtypes. A, *CTD-2015G9*.*2*. B, *CTD-2527I21*.*15*. C, *LINC00393*. D, *LINC01198*. E, *RP11-10A14*.*5*. F, *RP11-19E11*.*1*. Boxplots representing the basal-like subtype are highlighted in either red (UBCS) or blue (TCGA).

Of the nine lncRNAs defined as meta-genes by NMF in both the UBCS and TCGA basal-like clusters, six were also significantly over-expressed (log_2_ FC>1.00; adjusted *p*<0.05) in basal-like compared to other breast cancer subtypes (Table 2; Fig 2; S5a and S5b Table). We therefore classed these six genes as candidate lncRNA markers of basal-like breast cancer.

**Table 2:** A consensus list of lncRNAs associated with the basal-like breast cancer intrinsic subtype.

Ensembl ID	Gene Symbol	UBCS			TCGA		
		Log_2_ FC	*p*-value	Adjusted *p*-value	Log_2_ FC	*p*-value	Adjusted *p*-value
ENSG00000261175	*CTD-2015G9*.*2*	1.62	1.47E-07	7.28E-06	2.40	5.34E-98	2.28E-94
ENSG00000179066	*CTD-2527I21*.*15*	1.38	2.33E-15	5.44E-12	1.45	1.04E-65	6.68E-63
ENSG00000224853	*LINC00393*	1.90	1.45E-09	1.80E-07	1.50	4.22E-41	4.31E-39
ENSG00000231817	*LINC01198*	1.31	1.77E-03	1.16E-02	1.37	3.06E-50	7.48E-48
ENSG00000248538	*RP11-10A14*.*5*	1.91	6.03E-10	8.78E-08	1.67	6.40E-35	3.50E-33
ENSG00000258910	*RP11-19E11*.*1*	3.23	7.40E-24	1.90E-19	2.40	3.39E-80	4.35E-77

For the majority of markers, there was a clear distinction between high expression levels in basal-like tumours compared to other subtypes (Fig 2). Exceptions were *CTD-2015G9*.*2* (Fig 2A) and *LINC01198* (Fig 2D), both of which are also expressed in the normal-like tumours. Furthermore, expression was typically low across an extra 19 ER-/PR-/HER2+ TCGA tumours classified as HER2-enriched TCGA tumours compared to basal-like tumours (S1 Fig).

### Relationship between lncRNA basal-like breast cancer markers and neighbouring genes

Recent observations have shown that *cis*-acting lncRNAs tend to correlate strongly with their neighbouring genes [22]. Therefore, to determine potential *cis*-regulatory functions, we first defined neighbouring genes of each potential basal-like breast cancer lncRNA marker (Table 2) using GREAT [23] with the “basal plus extension” setting, and then calculated the Pearson correlation coefficient (*r*) between each lncRNA expression profile and its neighbouring genes across the TCGA breast cancer cohort. We repeated this calculation across all other cancer types represented in the TCGA in which the lncRNA achieved expression ((mean FPKM+SD)>1.00) and variability (CV>0.10) thresholds. We sought consistently high correlation across a number of cancer types as support for a potential *cis*-regulatory relationship.

**Fig 3:**
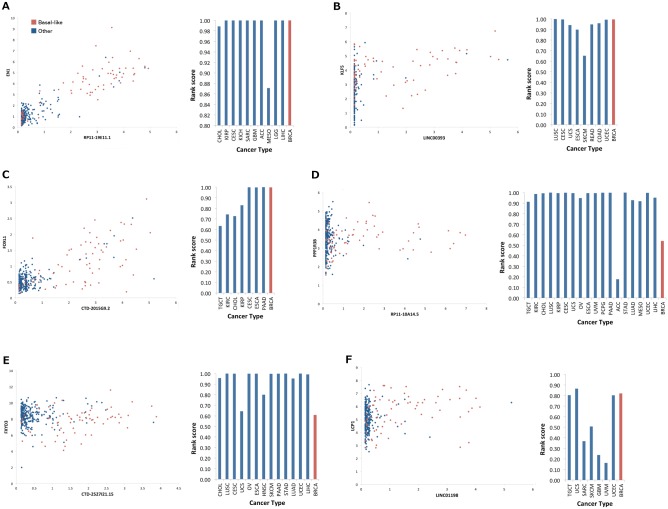
Correlation between six potential lncRNA basal-like breast cancer markers and their neighbouring genes, and comparison of correlation across cancer types. Each segment of the figure consists of (1) a scatterplot comparing FPKM expression values of the lncRNA with the neighbouring PC gene of interest, and (2) a comparison of the rank achieved by Pearson correlation of the PC gene with the lncRNA between cancer types. A, *RP11-19E11*.*1* versus *EN1*. B, *LINC00393* versus *KLF5*. C, *CTD-2015G9*.*2* versus *FOXL1*. D, *RP11-10A14*.*5* versus *PPP1R3B*. E, *CTD-2527I21*.*15* versus *FXYD3*. F, *LINC01198* versus *LCP1*. Only cancer types in which lncRNA achieves expression ((mean FPKM+SD)>1.00) and variability (CV>0.10) thresholds were considered. Rank score = 1-(n/N) where n = position of PC gene in list of PC genes ranked in descending order of correlation to lncRNA, and N = total number of PC genes (17308). Rank score>~0.99 indicates PC gene ranked in top 200. Breast cancer is highlighted in red. TCGA cancer type codes are listed in S8 Table.

Three of the six markers (*RP11-19E11*.*1*, *LINC00393* and *CTD-2015G9*.*2*) achieved significant correlation (*p*<0.0001) with a neighbouring gene (Table 3; Fig 3A–3C; S6 Table) across TCGA breast cancer tumours. Of note was the high correlation achieved between *RP11-19E11*.*1* and the transcription factor engrailed 1 (*EN1*; *r* = 0.90; Fig 3A). *EN1* is over-expressed in basal-like breast cancer [24], and achieved significant differential expression between basal and other breast cancers in both UBCS (log_2_FC = 4.03, *p* = 2.26E-22) and TCGA (log_2_FC = 3.01, *p* = 8.04E-71) datasets. *EN1* is also consistently correlated with *RP11-19E11*.*1* across multiple cancers, achieving the highest correlation amongst 17308 PC genes in 7/11 cancers (Fig 3A; S7a Table). Similarly, *LINC00393* achieved significant correlation with the transcription factor krueppel-like factor 5 (*KLF5*; *r* = 0.45; Fig 3B), whose high expression in basal-like breast cancers [25] was supported by both UBCS (log_2_FC = 2.18, *p* = 6.52E-05) and TCGA (log_2_FC = 1.73, *p* = 1.07E-30) datasets. Its correlation with *LINC00393* also ranked highly in lung squamous cancer compared to other PC genes (Fig 3B; S7b Table). The strong correlation between *CTD-2015G9*.*2* and *FOXL1* forkhead box L1 (*FOXL1*) in breast cancer (*r* = 0.73; Fig 3C) was repeated across 4/8 cancers (S7c Table). *FOXL1* has not been reported as a TN or basal breast cancer marker, although other members of the forkhead family of transcription factors such as *FOXA1* (UBCS: log_2_FC = -5.04, *p* = 4.05E-18; TCGA: log_2_FC = -4.89, *p* = 9.81E-136) and *FOXC1* (UBCS: log_2_FC = 2.58, *p* = 3.43E-15; TCGA: log_2_FC = 3.16, *p* = 5.83E-103) are established regulators of luminal and basal-like breast cancers respectively. *FOXL1* was over-expressed in both datasets used in this study (UBCS: log_2_FC = 0.50, *p* = 9.18E-07; TCGA: log_2_FC = 0.75, *p* = 2.50E-42) albeit with relatively small fold changes.

**Table 3:** Correlations between basal lncRNA markers and their neighbouring genes.

LncRNA	Neighbour^a^	Pearson correlation		
		All (349 samples)	Non-basal (271)	Basal only (68)
*CTD-2015G9*.*2*	*FOXL1*	0.73*	0.26*	0.50*
*CTD-2527I21*.*15*	*FXYD3*	-0.02	0.07	0.52*
*LINC00393*	*KLF5*	0.45*	0.08	0.26
*LINC01198*	*LCP1*	0.25	0.06	0.21
*RP11-10A14*.*5*	*PPP1R3B*	-0.03	-0.06	0.29
*RP11-19E11*.*1*	*EN1*	0.90*	0.52*	0.80*

^a^Neighbouring PC gene achieving highest correlation

*Pearson *p*-value<0.0001

By contrast, no significant correlation was observed between the three remaining lncRNAs, *CTD-2527I21*.*15*, *RP11-10A14*.*5* and *LINC00198*, and their neighbouring genes across breast cancer (Fig 3D–3F), although there was evidence for a *cis*-regulatory role across other cancers. For example, protein phosphatase 1 regulatory subunit 3B (*PPP1R3B*) was ranked in the top 100 most correlated PC genes with *RP11-10A14*.*5* in 10/19 cancers (Fig 3D; S7d Table), and FXYD domain containing ion transport regulator 3 (*FXYD3*) with *CTD-2527I21*.*15* in 8/14 cancers (Fig 3E; S7e Table). Interestingly, a higher correlation across basal-like compared to other breast cancers was observed for both *PPP1R3B* (basal-like: *r* = 0.29, other: -0.06) and *FXYD3* (basal-like: *r* = 0.52, other: 0.07), significant at *p*<0.01 for *FXYD3*, suggesting their *cis*-regulatory role is restricted to the basal-like subtype. A similar pattern was observed between *LINC01198* and its nearby PC gene *LCP1*, where correlation increased from *r* = 0.06 across non-basal-like to *r* = 0.21 across basal-like breast cancers, although this was not significant and there was no evidence of *cis*-regulation of *LCP1* by *LINC01198* in other cancer types (Fig 3F; S7f Table).

### Identification of clusters associated with the tumour microenvironment

To understand the poor overlap between ER+ luminal A clusters, and characterize the mixed subtype cluster of UBCS, we functionally profiled these clusters from both TCGA and UBCS using the guilt-by-association approach [14]. Briefly, the Pearson correlation coefficient (*r*) was calculated between each member of the lncRNA meta-gene and the 11027 and 12410 PC genes achieving the detection ((mean FPKM+SD)>1.00) and variability (CV>0.10) thresholds across UBCS and TCGA respectively. PC genes achieving *r*>0.60 (UBCS) or *r*>0.50 (TCGA) were then input to DAVID functional enrichment analysis [26].

The majority (9/14) of lncRNA meta-genes of the UBCS mixed subtype cluster were associated with the immune response or related processes (S9a Table). 8/20 lncRNAs of the TCGA ER+ luminal A cluster were associated with the extra-cellular matrix (S9b Table), and all achieved significant correlation with at least one of two established cancer-associated fibroblast (CAF) markers: fibroblast activation protein (*FAP*) and actin, alpha 2, smooth muscle, aorta (*ACTA2*; S9b Table). By contrast, no significant correlation was observed between these two genes and any of the lncRNAs related to the immune response.

**Fig 4:**
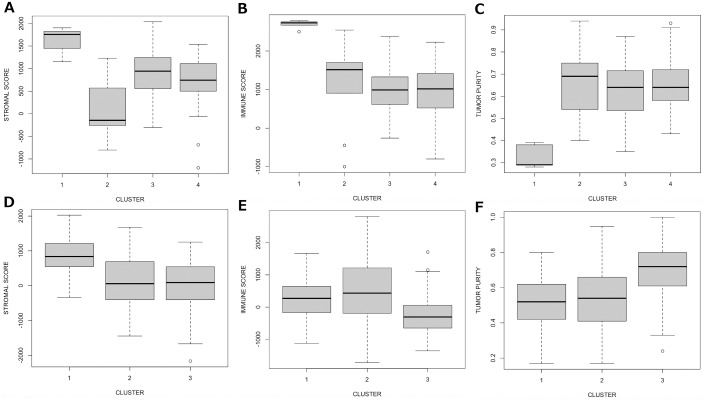
Stromal cell, immune cell and tumour purity measures for each cluster derived from UBCS and TCGA samples according to ESTIMATE [27]. UBCS: A, stromal cell content. B, immune cell content. C, tumour purity. TCGA: D, stromal cell content. E, immune cell content. F, tumour purity.

These findings were supported by ESTIMATE [27] prediction of tumour purity across each cluster. High stromal (Fig 4A) and immune cell (Fig 4B) content was observed in samples of the UBSC mixed subtype cluster and this contributed significantly to their low tumour purity (0.33±0.05; *p*<0.001 by T-test) compared to the other three clusters (Fig 4C). By contrast, high stromal cell content was observed in some ER+ luminal A TCGA samples (Fig 4D) but there was no significant difference in immune cell score (Fig 4E) or tumour purity (Fig 4F) between the clusters. Overall, only 1.4% (5/339) TCGA samples achieved immune cell ESTIMATE score>2000 compared to 11/63 (17.4%) of UBCS samples. Neither sample cohort had been subject to micro-dissection to separate the tumour from non-tumour cells.

We next checked for evidence of expression in samples expected to consist of exclusively tumour cells, reasoning that lncRNAs expressed in clinical samples that typically contain a proportion of non-tumour cells (TCGA: mean tumour purity = 0.60±0.17, UBCS: 0.62±0.15), but with little or no expression in samples of high tumour cell purity, were likely stromal or immune cell specific. To do so, we calculated lncRNA expression levels across 41 breast cancer cell lines from the Cancer Cell Line Encyclopaedia [28] (CCLE; mean tumour purity = 0.99±0.01), and tumours from 10 breast cancer patient derived xenograft (PDX) models [29] (mean tumour purity = 0.99±0.01), in which tumour had been separated from stroma using an *in silico* species-specific mapping strategy [30]. For 5219 lncRNAs common to all three datasets, we then compared both median cell line and PDX expression to median expression across 47 UBCS samples achieving tumour purity>0.70.

Note that the three datasets were generated using different sequencing protocols and so subject to a number of confounding factors. Therefore this was not intended as a rigorous statistical assessment, rather a conservative guide to genes consistently over-expressed in cell lines and the tumour component of PDX models compared to clinical samples.

198 lncRNAs achieved log_2_FC>0.50 in both comparisons, and median FPKM<0.50 across cell lines and PDX tumours (S10 Table), thus were classed a potentially stromal or immune cell specific (SIC). Included in this list was the maternally expressed 3 gene (*MEG3*), one of the few lncRNAs known to be preferentially expressed in tumour stroma [31]. This achieved the highest fold changes in both cell lines (log_2_FC = 6.74) and the PDX tumour component (log_2_FC = 6.73), adding confidence to our approach. We observed the greatest overlap between SIC lncRNAs and the UBCS mixed subtype (7/14; 43%) and TCGA ER+ luminal A (7/20; 35%) clusters. No overlap was achieved with the basal-like clusters, and only some overlap with UBCS (3/28; 11%) and TCGA luminal A/B (2/33; 6%) clusters, and UBCS ER+ luminal A cluster (2/12; 17%). SIC lncRNAs indicated by this method are listed in S11 Table.

### A putative set of breast cancer stromal and immune cell associated lncRNAs

By combining evidence from the guilt-by-association and cell line/PDX expression profiling, we derived a set of four and six lncRNAs achieving functional enrichment FDR<0.05 for “immune response” or “extracellular matrix” respectively, and low/undetectable expression in cell lines/PDX tumour (Table 4; S12 Table). All extracellular matrix associated lncRNAs achieved significant Pearson correlation with *FAP* and *ACTA2* (Table 4b; S9b Table), suggesting a potential role in activating fibroblasts. For comparison, we also carried out the same analyses on known stromal lncRNA *MEG3* [31], which achieved significant (*p*<0.0001) correlation with both *FAP* (*r* = 0.63) and *ACTA2* (*r* = 0.53), and enrichment for “extracellular matrix” (*p* = 8.88E-38). Consequently, *MEG3* met all criteria for our classification of a stromal cell associated lncRNA.

**Table 4:** LncRNAs associated with breast cancer stromal or immune cells.

Ensembl ID	Gene symbol	Functional enrichment		Co-expression	
		Top enrichment	*p*-value	*FAP*	*ACTA2*
(a) Immune cell associated^a^					
ENSG00000234147	*RP3-460G2*.*2*	Immune response	1.06E-09	0.36	0.3
ENSG00000259225	*RP11-1008C21*.*1*	Immune response	1.53E-25	0.21	0.17
ENSG00000273341	*RP5-899E9*.*1*	Immune response	1.30E-16	0.32	0.21
(b) Stromal cell associated^b^					
ENSG00000261742	*LINC00922*	Extracellular matrix	1.26E-46	0.64*	0.41*
ENSG00000254366	*RP11-38H17*.*1*	Extracellular matrix	2.01E-11	0.44*	0.35*
ENSG00000232679	*RP11-400N13*.*3*	Extracellular matrix	1.30E-31	0.62*	0.37*
ENSG00000261039	*RP11-417E7*.*2*	Extracellular matrix	1.46E-57	0.78*	0.57*
ENSG00000261327	*RP11-863P13*.*3*	Extracellular matrix	8.03E-47	0.75*	0.50*
ENSG00000233521	*RP5-1172A22*.*1*	Extracellular matrix	4.59E-49	0.71*	0.48*

^a^*r*-values calculated across 63 samples, *r*>0.60 used as threshold for enrichment gene list.

^b^*r*-values calculated across 1093 samples, *r*>0.50 used as threshold for enrichment gene list.

*Pearson *p*-value<0.0001

**Fig 5:**
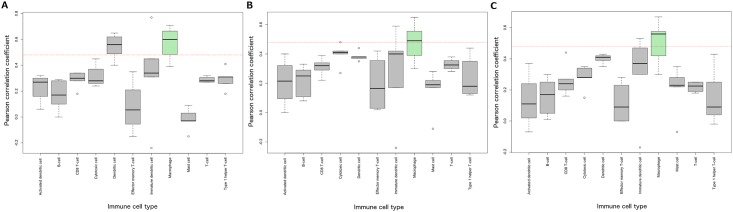
Correlation of immune response-associated lncRNAs with specific immune cell type. A, *RP11-1008C21*.*1*. B, *RP5-899E9*.*1*. C, *RP3-460G2*.*2*. Each box is generated from correlations obtained between each lncRNA and a set of established markers for the corresponding immune cell type. Red horizontal dashed line at *r* = 0.48 indicates significance at *p*<0.0001. The macrophage cell type achieves median *r*>0.48 across all lncRNAs and is highlighted in green. Only immune cell types represented by >3 markers are shown on the boxplots.

We next explored whether the three putative immune-response associated lncRNAs were linked with a specific immune cell type. To do so, we selected a set of established markers for immune cell type and calculated the correlation between each marker and the lncRNA (S12 Table). For cell types represented by at least four markers, the median correlations achieved by each cell type were compared (Fig 5). All three immune cell associated lncRNAs achieved the strongest correlations with macrophage cell type markers such as *CD68* (*RP3-460G2*.*2*: *r* = 0.67, *RP11-1008C21*.*1*: *r* = 0.68, *RP5-899E9*.*1*: *r* = 0.65) and macrophage scavenger receptor 1 (*MSR1*; *RP3-460G2*.*2*: *r* = 0.56, *RP11-1008C21*.*1*: *r* = 0.67, *RP5-899E9*.*1*: *r* = 0.52). No other cell type achieved a median *r*>0.48 (*p*<0.0001) across all three lncRNAs.

## Discussion

In this study, we first establish that lncRNA expression alone is sufficient to separate breast cancer into clusters that broadly correspond to intrinsic subtype. This enabled us to identify a consensus set of lncRNAs associated with the basal-like breast cancer, and generate hypotheses on their relationship with neighbouring genes. Secondly, we find evidence that the lack of agreement between signatures derived from other subtypes is a result of the varying degree of stromal/immune cell infiltrate present in a typical clinical sample. Finally, we derive a set of lncRNAs whose expression is specific to the breast tumour microenvironment, with possible roles in activating fibroblasts to support tumour progression, and macrophage recruitment to the tumour.

### A consensus set of basal-like breast cancer lncRNA markers and their potential cis-regulatory roles

We derived a set of six high confidence lncRNA basal-like markers, all of which were significantly over-expressed in basal-like tumours from both UBCS and TCGA, and made a significant contribution to the basal-like NMF clusters. Basal-like breast cancers are characterised by aggressive features and frequently associated with poor prognosis. They account for 85% of TNBCs, which lack expression of oestrogen, progesterone or HER2 receptors and as such, fail to respond to hormone targeting therapies. With rigorous follow-up, our lncRNA markers could indicate novel regulatory mechanisms specific to basal-like breast cancer, providing a platform to generate new drug targets.

As an initial exploration, three of these (*RP11-19E11*.*1*, *CTD-2015G9*.*2*, *LINC00393*) achieved significant expression correlation with a neighbouring PC gene, suggesting a possible *cis*-regulatory relationship. Notably, PC genes *EN1* and *KLF5* neighbouring *RP11-19E11*.*1* and *LINC00393* respectively are known markers of the basal-like breast cancer subtype [24][25]. In all three cases, the relationship was not restricted to breast cancer but extended across at least one other cancer type, and for *RP11-19E11*.*1* and *CTD-2015G9*.*2* across at least half the cancer types in which expression of the lncRNA could be detected. To our knowledge, this represents a unique application of the guilt-by-association approach to identify *cis*-regulation through consistent pan-cancer co-expression between lncRNAs and their neighbouring genes. By doing so, we may have uncovered a potential route by which these lncRNAs control important basal-like PC genes.

For two of the remaining lncRNA-neighbouring PC gene pairs (*CTD-2527I21*.*15* and *FXYD3*, *RP11-10A14*.*5* and *PPP1R3B*) co-expression only emerged across basal-like tumours despite consistent pan-cancer correlation. The greatest increase was seen between *CTD-2527I21*.*15* and *FXYD3*, a gene not previously associated with basal-like breast cancer but whose expression has been shown to increase in response to oestrogen and tamoxifen [32]. Our results demonstrate the need to consider disease subtype specificity when seeking transcriptional evidence of *cis*-regulation by lncRNAs.

### Tumour purity confounds clustering but identifies tumour microenvironment-associated lncRNAs

Our finding that lncRNA expression clusters broadly correspond to intrinsic molecular subtype is in accordance with previous studies [12][17][18]. Clear enrichment was observed for ER+ luminal A, ER+ luminal A/B and TNBC/ER+ basal-like subtypes in three of the clusters from both datasets. However, the fourth UBCS cluster comprised a mix of subtypes and showed no clear correspondence to a TCGA cluster. Notably, this cluster appeared to be driven by lncRNAs associated with the immune response, concordant with a greater proportion of samples with predicted high immune cell content in the UBCS cohort than TCGA. We also found that the ER+ luminal A cluster derived from TCGA is driven partially by lncRNAs associated with tumour stroma, a trend not observed in the equivalent UBCS cluster. Our results suggest that signature inconsistency is in part driven by the varying extent of stromal and immune cell infiltrate in patient samples, supporting a recent study that determined the confounding effects of tumour purity on differential expression and co-expression measurements [33].

The presence of non-tumour cells highlighted a small set of lncRNAs whose expression is restricted to the tumour microenvironment. The tumour microenvironment consists of multiple cell types including endothelial cells, adipocytes, CAFs and immune cells such as lymphocytes and tumour-associated macrophages that play a critical role in supporting cancer growth and metastasis [34]. An association between lncRNAs and the immune response has only recently emerged [35][36], and only a few have been shown to be expressed in endothelial cells [37], and adipocytes [38], with preferential expression of *MEG3* [31] and *H19* [31][39] observed in tumour stroma.

The observation that our putative immune cell associated lncRNAs correlate strongly with macrophage markers is consistent with tumour-associated macrophages (TAMs) as the most abundant immunosuppressive cell population in breast tumours. TAMs are frequently associated with poor prognosis [40], and their relationship with tumour cells is currently under intense scrutiny since the disruption of the positive-feedback loop between TAMs and breast cancer cells could inhibit the angiogenic and/or metastatic potential of the tumour. Therefore, lncRNAs could offer a novel avenue to target the TAM population, and supplement immunotherapy.

The high correlation between the six extracellular matrix-associated lncRNAs with CAF markers *FAP* and *ACTA2* may suggest a role for lncRNAs in the acquisition of an activated phenotype by fibroblasts in the tumour stroma. Currently, the mechanisms of fibroblast activation are poorly understood, and so the possibility that numerous lncRNAs may have a role in their regulation opens up an enticing research opportunity. Overall, our discovery of several lncRNAs specifically expressed in either stromal or immune cells should stimulate further studies to determine their precise role in the tumour life-cycle, potentially leading to novel therapeutic strategies.

## Conclusions

We have performed a comprehensive analysis of lncRNA in breast cancer that builds on previous findings that lncRNA expression alone is sufficient to separate breast cancer into clusters that broadly correspond to hormone status and intrinsic molecular subtype. By combining two independent clinical datasets, we establish a set of lncRNA markers specific to basal-like breast cancer, representing a preliminary effort to exploit the disease subtype specificity of lncRNAs. With rigorous validation, at least a subset of these could have clinical potential as either biomarkers or therapeutic targets. We also demonstrate the confounding effects of tumour purity as a source of inconsistency in signatures derived from different studies. The presence of non-tumour cells in our patient samples provided the opportunity to discover several lncRNAs specifically expressed in the tumour microenvironment. Our list should motivate follow-up studies to establish whether these lncRNAs are key regulators of either macrophage recruitment or fibroblast activation, and thus critical in supporting tumour growth and progression.

## Methods

### RNA-Seq sample preparation and data processing

#### Utah Breast Cancer Study (UBCS)

Fresh frozen breast tissue samples were obtained from 88 women who had surgery at the Huntsman Cancer Hospital from 2009–2012, including tumour tissues from 69 breast cancer patients. One tumour sample yielded poor quality RNA (RIN = 2.5) and was removed from consideration, resulting in a panel of 68 tumour samples. RNA libraries were made with the Illumina TruSeq Stranded mRNA Sample Preparation kit with oligo dT selection according to the manufacturer's protocol. These libraries were then submitted for 50bp single-end sequencing on the Illumina HiSeq 2000 platform using eight samples per lane. For the purposes of this analysis, five breast cancers of the HER2 subtype and one of ambiguous hormone receptor status were ignored. The reads for the remaining 63 were aligned to the human (GRCh38) genome using StarAlign [41] with no more than three mismatches and only uniquely mapped reads allowed. Reads whose ratio of mismatches to mapped length was greater than 0.10 were also discarded. All other parameters were set to their defaults for stranded alignment. Mapped read counts were consistent (14M-23M) across samples, so no samples were removed due to low mapping rate (S1 Table). The expression level, based on Fragments Per Kilobase per Million fragments mapped (FPKM), of each gene present in the human (GRCh38) GENCODEv22 annotation file was estimated using Cufflinks with library type defined as “fr-firststrand” and all other parameters set to defaults [42]. Only genes annotated as “lincRNA” or “protein_coding” were considered. LncRNAs overlapping PC genes were ignored for consistency with the TCGA dataset, as well as genes whose largest transcript is less that 400bp due to potential over-estimation of expression across transcripts less than the average fragment length. The resulting gene-by-sample matrix consisted 19567 protein-coding genes and 6062 lncRNAs across 53 non-basal and 10 basal-like samples. Intrinsic subtypes were assigned according to PAM50 classification [43]. Differentially expressed genes (|log_2_FC|>1.5 and FDR<0.05) were identified using Limma [44] with eBayes function parameter “trend” set to “TRUE” and all other parameters set to their default values. Raw sequence data associated with UBCS have been deposited in the ArrayExpress database (www.ebi.ac.uk/arrayexpress) under accession number E-MTAB-4993.

#### The Cancer Genome Atlas (TCGA)

Raw FASTQ solid tumour sequence files from 1136 breast cancer patients were downloaded from the Cancer Genomics Hub (CGHub; https://cghub.ucsc.edu/), and reads aligned to the human (GRCh38) genome using the same procedure as for UBCS except all parameters were set to their defaults for un-stranded alignment. To reduce possible biases introduced by variable total read counts between samples, tumours achieving <20,000,000 mapped reads were removed. FPKM values for each gene present in the human (GRCh38) GENCODEv22 annotation file were calculated as before using Cufflinks with library type defined as “fr-unstranded” [42], and then batch normalized using COMBAT [45]. Sequencing data for all other TCGA cancer types used in this study were processed using the same procedure. The number of tumours used across each cancer type is given in S8 Table.

Only tumours classified as either ER+ or TNBC according to TCGA Network [46] were considered in subsequent analyses, and breast cancer samples treated with tamoxifen were discarded. The resulting matrix consisted of 19567 PC genes and 6062 lncRNAs across 271 non-basal and 68 basal-like samples. Differentially expressed genes were identified using Limma as for the UBCS dataset.

#### Cancer Cell Line Encyclopaedia (CCLE)

BAM files consisting of reads mapped to the human (GRCh37) genome were downloaded from the Cancer Genomics Hub (CGHub; https://cghub.ucsc.edu/) for all breast cancer cell lines represented in the CCLE [28], except those of the claudin-low subtype according to [47], or with fibroblast morphology according to ATCC (http://www.lgcstandards-atcc.org). FPKM values for each gene present in the human (GRCh38) GENCODEv19 annotation file were calculated as before using Cufflinks with library type defined as “fr-unstranded”. Of the 6995 lncRNAs annotated in GENCODEv19, 5284 overlapped with v22 based on Ensembl identifier. 38 overlapped by gene name only but were discarded since an Ensembl identifier change indicates that the gene structure has changed significantly between releases. Therefore, the resulting gene-by-sample matrix consisted of 5284 lncRNAs across 41 cell lines.

### Clustering of gene expression data with consensus non-negative matrix factorization (NMF)

We applied NMF to cluster breast cancer lncRNA transcriptomes from UBCS and TCGA. Only the most highly expressed and variable lncRNAs were chosen for clustering according to the following criteria: (mean FPKM+SD)>1.00 and CV>0.10, where CV = coefficient of variation. The underlying principle of NMF is dimensionality reduction in which a small number of meta-genes, each defined as a positive linear combination of the genes in the expression data, are identified and then used to group samples into clusters based on the gene expression pattern of the samples as positive linear combinations of these meta-genes. Using the R package *NMF* [48], factorization rank *k* was chosen by computing the clustering for *k* = 2–6 against 50 random initializations of both the actual and a permuted gene expression matrix, and selecting the *k* value achieving the largest difference between cophenetic correlation coefficients calculated from the actual and permutated data (S1 Fig). For further visual confirmation of a sensible choice of *k*, consensus matrices were generated corresponding to different *k* values (S2 Fig). To achieve stability, the NMF algorithm was then run against 200 perturbations of each gene expression matrix at the chosen values of *k* = 4 (UBCS) and *k* = 3 (TCGA).

### Ethics statement

UBCS tissues were attained and studied under written informed consent, as approved by University of Utah Institutional Review Boards 10924 (Molecular Classifications of Cancer) and 38201 (Genetic Epidemiology of Breast Cancer)."

## Supporting Information

S1 Fig Comparison of lncRNA basal-like marker expression with 19 HER2 TCGA tumours. A, *CTD-2015G9*.*2*. B, *CTD-2527I21*.*15*. C, *LINC00393*. D, *LINC01198*. E, *RP11-10A14*.*5*. F, *RP11-19E11*.*1*.
(TIF)

S2 Fig Rank quality measures generated by the R package *NMF* [48]. Quality measures computed from 50 runs for each value of rank *k* across A, UBCS, and B, TCGA expression datasets.
(TIF)

S3 Fig Rank consensus matrices generated by the R package *NMF* [48]. Consensus matrices computed from 50 runs for each value of rank *k* across A, UBCS, and B, TCGA.
(TIF)

S1 Table UBCS sample details and sequencing statistics. (XLSX)

S2 Table TCGA sample details and sequencing statistics. (XLSX)

S3 Table NMF cluster meta-genes derived from (a) UBCS, and (b) TCGA. (XLSX)

S4 Table Number of samples clustered to breast cancer subtypes defined by hormone status and PAM50 in (a) UBCS, and (b) TCGA cohorts. (XLSX)

S5 Table Basal-like versus non-basal-like differential expression analysis across (a) UBCS, and (b) TCGA. (XLSX)

S6 Table Differential expression and correlation analyses across nearest neighbours of consensus lncRNA basal-like markers. (XLSX)

S7 Table Rank of correlation between each lncRNA basal-like marker and its nearest neighbours across cancer types. (XLSX)

S8 Table TCGA codes for each cancer type and the number of tumours used in the correlation analyses. (XLSX)

S9 Table Evidence of immune and stromal cell association of lncRNA meta-genes from (a) UBCS and (b) TCGA cluster one. (XLSX)

S10 Table List of 198 lncRNAs expressed in breast cancer patient samples but with undetectable/low expression in cell lines and tumour compartment of patient-derived xenograft models. (XLSX)

S11 Table List of lncRNA meta-genes from (a) UBCS, and (b) TCGA overlapping the 198 lncRNAs in S10 Table. (XLSX)

S12 Table Correlation between three putative immune cell-associated lncRNAs and immune-cell type specific expression markers. (XLSX)
