# Correction: Consensus Analysis of Whole Transcriptome Profiles from Two Breast Cancer Patient Cohorts Reveals Long Non-Coding RNAs Associated with Intrinsic Subtype and the Tumour Microenvironment

**DOI:** 10.1371/journal.pone.0192589

**Published:** 2018-02-06

**Authors:** James R. Bradford, Angela Cox, Philip Bernard, Nicola J. Camp

There is information missing from the Competing interests statement. The Competing interests statement should read: Philip Bernard is an inventor of the PAM50 signature and a stakeholder in BioClassifier LLC, a company that licensed the PAM50 know-how to Nanostring Inc. for commercialization of Prosigna®. PB also has been a Medical Director at ARUP Laboratories Inc since 2003. This does not alter our adherence to PLOS ONE policies on sharing data and materials.
