# Supplementary figures and images for: Consensus Analysis of Whole Transcriptome Profiles from Two Breast Cancer Patient Cohorts Reveals Long Non-Coding RNAs Associated with Intrinsic Subtype and the Tumour Microenvironment
Source: PLoS One. 2016 Sep 29;11(9):e0163238. doi: 10.1371/journal.pone.0163238 (PMC5042460; doi:10.1371/journal.pone.0163238)

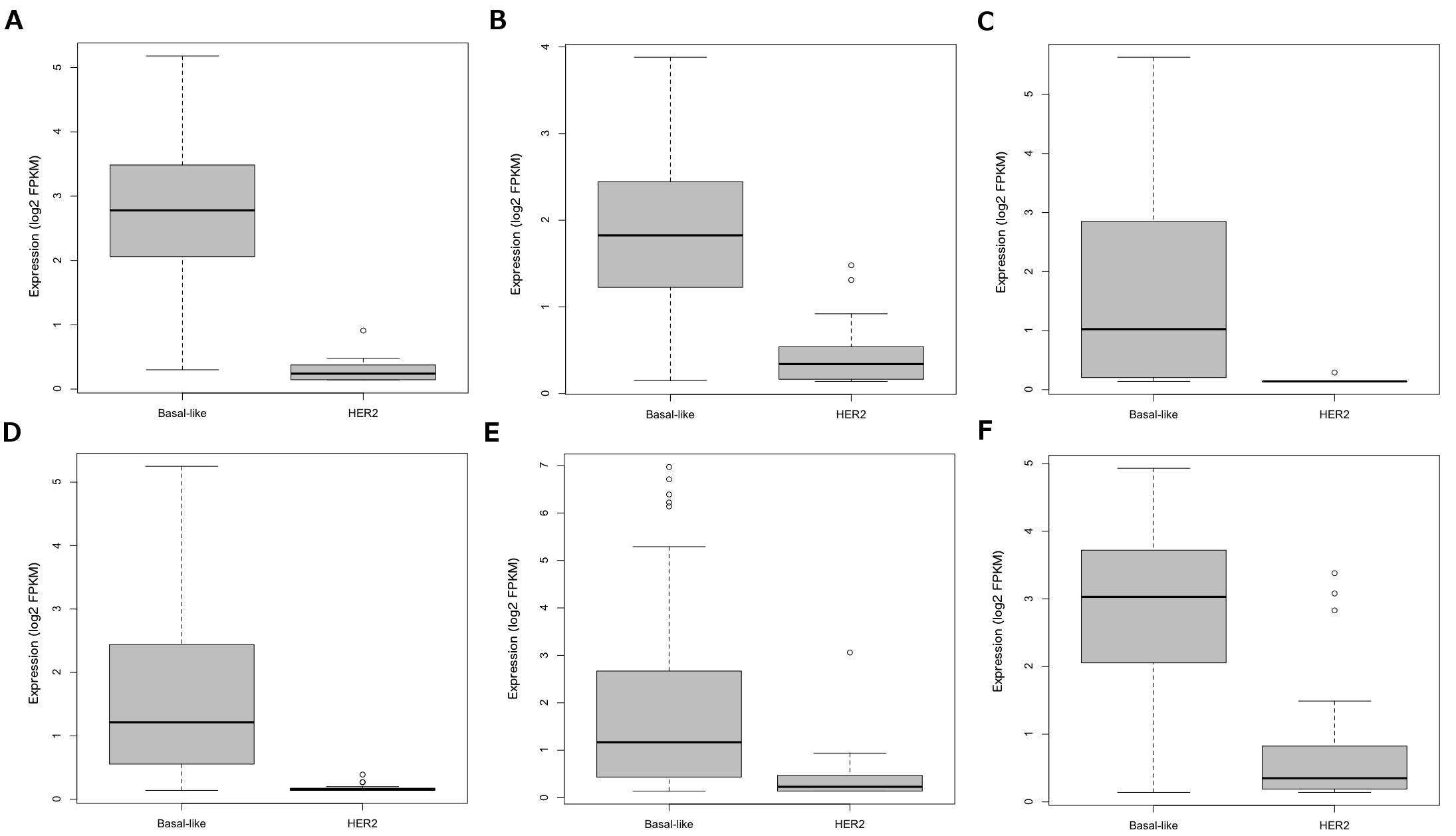

Supplement: S1 Fig — A, CTD-2015G9.2. B, CTD-2527I21.15. C, LINC00393. D, LINC01198. E, RP11-10A14.5. F, RP11-19E11.1. (TIF) [file pone.0163238.s001.tif]

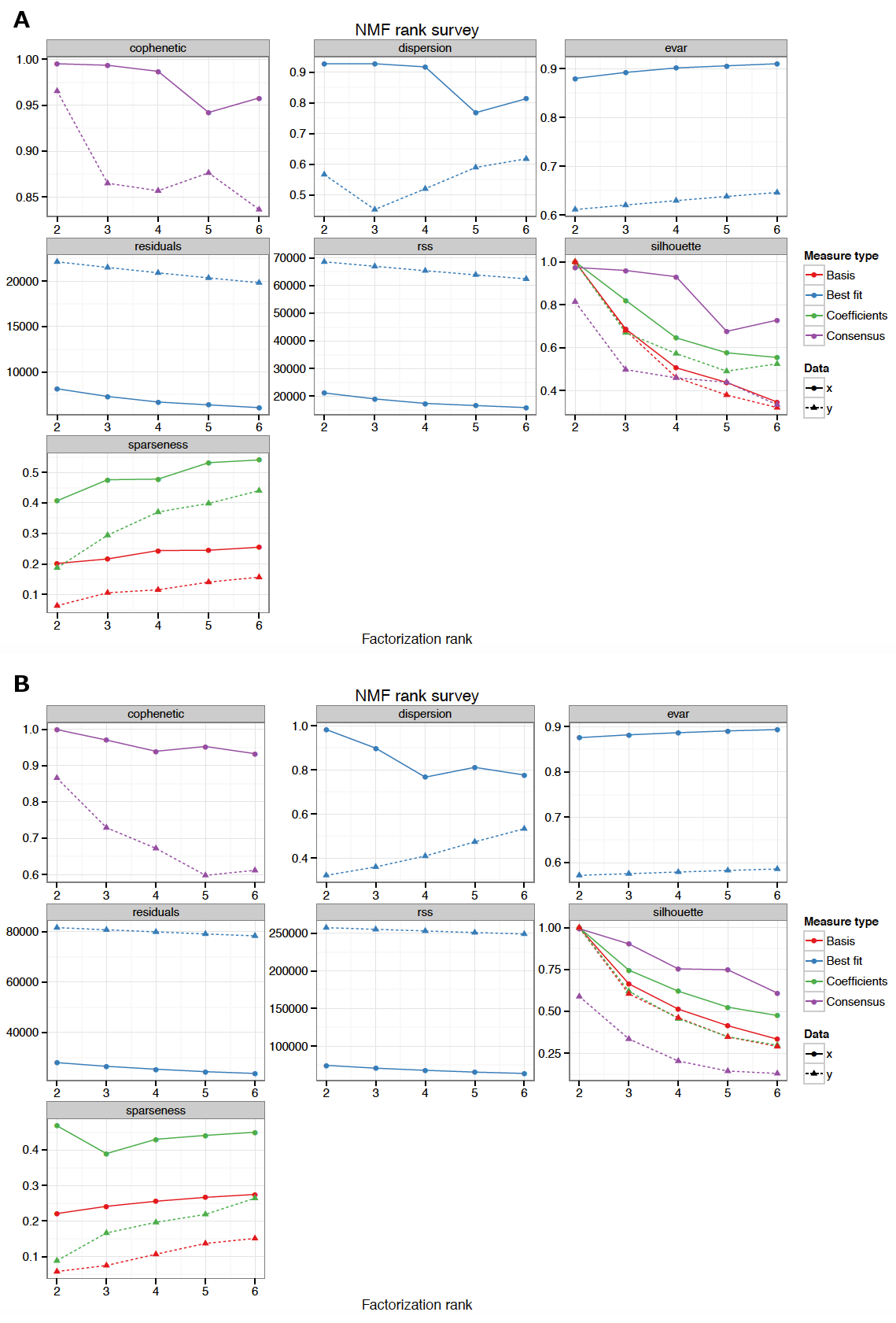

Supplement: S2 Fig — Quality measures computed from 50 runs for each value of rank k across A, UBCS, and B, TCGA expression datasets. (TIF) [file pone.0163238.s002.tif]

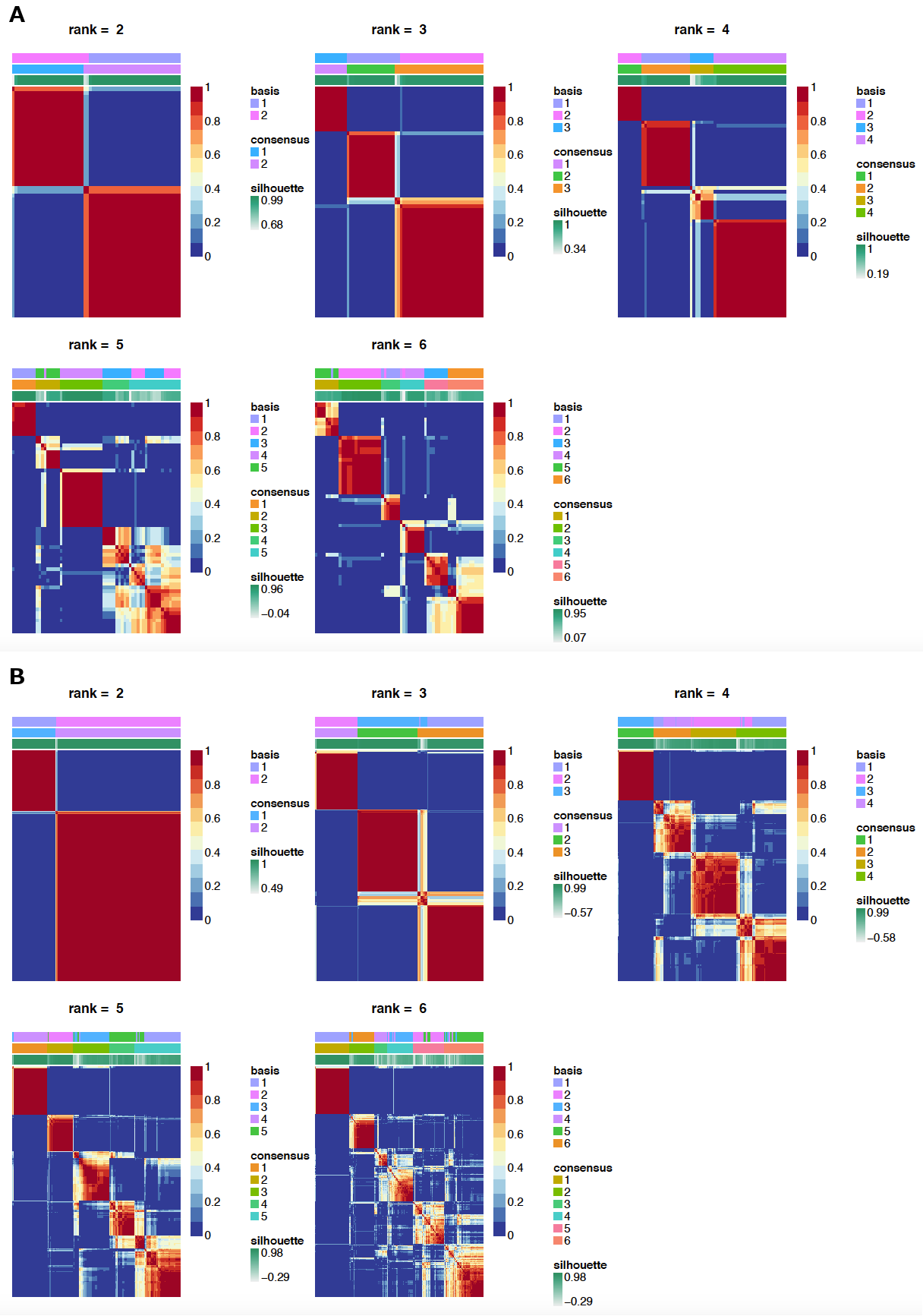

Supplement: S3 Fig — Consensus matrices computed from 50 runs for each value of rank k across A, UBCS, and B, TCGA. (TIF) [file pone.0163238.s003.tif]
